# Combined Therapy for Distant Metastasis of Sacral Chordoma

**DOI:** 10.1155/2015/165162

**Published:** 2015-01-11

**Authors:** Birol Özkal, Can Yaldız, Peyker Temiz, Cüneyt Temiz

**Affiliations:** ^1^Department of Neurosurgery, Alanya Government Hospital, 07400 Alanya, Turkey; ^2^Department of Neurosurgery, Sakarya Training and Research Hospital, 54100 Sakarya, Turkey; ^3^Department of Pathology, Medical Faculty, Celal Bayar University, 45030 Manisa, Turkey; ^4^Department of Neurosurgery, Medical Faculty, Celal Bayar University, 45030 Manisa, Turkey

## Abstract

Chordomas are known as rare primary malign tumours that have formed from primitive notochord remains. Sacral chordomas grow slowly but locally and aggressively. Chordomas are locally invasive and have low tendency to metastasis and have a poor prognosis in long-term follow-up. Metastasis may be seen in a rate of 5–40% of the chordomas. Metastasis of chordomas is common in liver, lung, lymph nodes, peritoneum, and brain. The treatment approaches, including surgery, have been discussed in the literature before. Susceptibility to radiotherapy and chemotherapy is controversial in these tumours. The success of surgical treatment affects survival directly. In this report, we will report a sacral chordoma case in which an intraperitoneal distant metastasis occurred and discuss the surgical approach.

## 1. Objective

Chordomas are low-grade, rare, and primarily malign tumors that grow slowly. They rarely have distant metastasis, but instead more locally recurrent tumors grow from primitive notochordal cell remnants. The yearly incidence is estimated at 5/1,000,000; 50–60% of cases are seen in the sacrum, 25–35% at the skull base, and 10–15% in the thoracolumbar region [1–5].

In this study, we report a case of sacral chordoma in a 53-year-old male with a distant metastasis to the peritoneal region; such a case has never been published before. Preoperatively, the patient was examined for metastasis, and none was found. The mass was removed via the posterior approach. Additional adjuvant radiotherapy was applied to the sacrococcygeal region. At early postoperative evaluation (one and three months), no local recurrence or distant metastasis was found. However, at the ninth month postoperatively, intraperitoneal distant metastases were seen and all metastases were completely extirpated by a general-surgery team. Postoperatively, conformal complex radiotherapy was applied. There was no metastasis or local recurrence within one year following the final surgery.

## 2. Case

**Figure 1:**
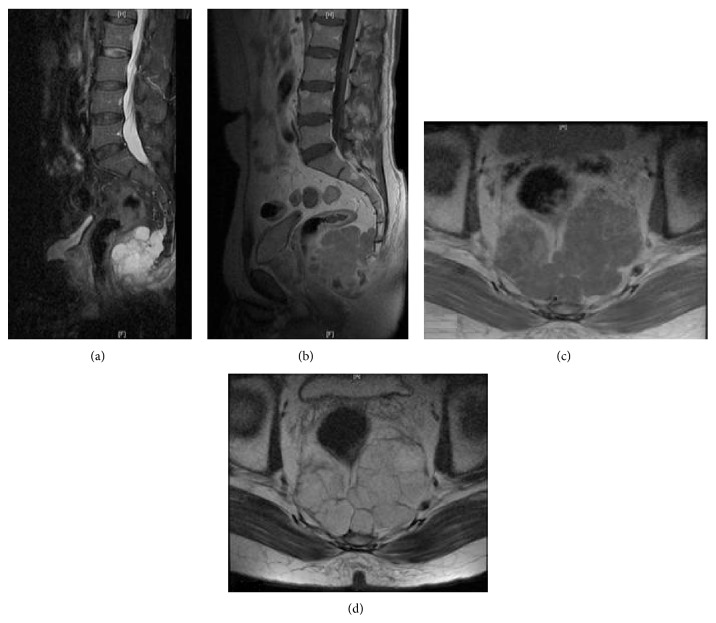
Preoperative sagittal and axial MRI of sacrococcygeal chordoma.

**Figure 2:**
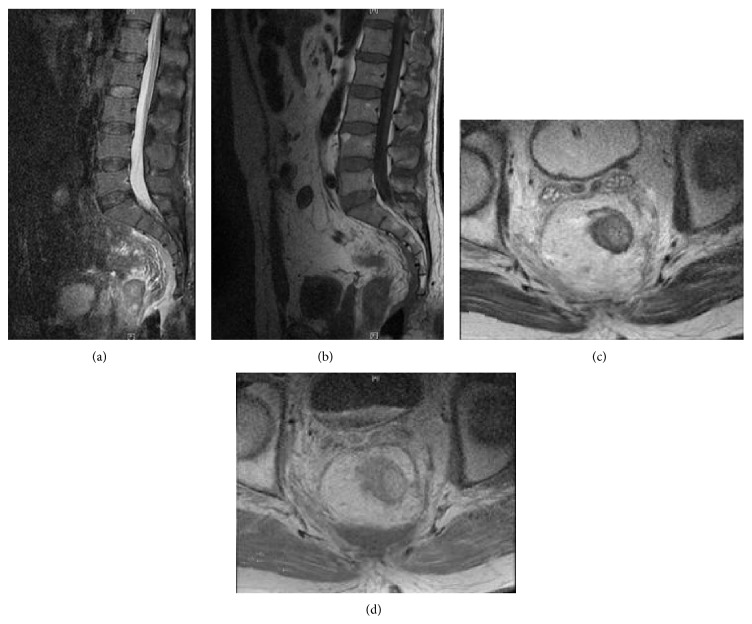
Sagittal and axial MRI following the removal of the mass.

**Figure 3:**
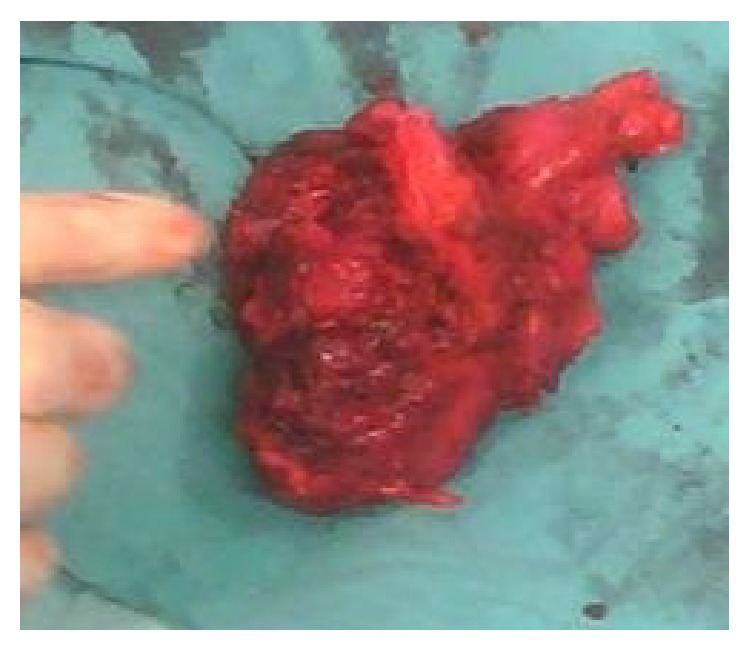
Macroscopic appearance of the mass.

A 53-year-old male was referred to our clinic with coccydynia and coccygeal effluence due to a local mass. Complaints had started three months previously. No neurological symptoms were evidenced in the neurological examination, and there was no urinary or fecal retention. Magnetic resonance imaging (MRI) of the sacrococcygeal spine showed a well-defined lobulated mass in the sacrococcygeal region (Figure 1). The sacrococcygeal mass was completely removed via retroperitoneal posterior approach (Figures 2 and 3). No neurological deficit was observed after surgery. Postoperative MRI showed total excision of the tumor and no local or distant metastasis. The specimens were histopathologically diagnosed as chordoma. Conformal complex radiotherapy was applied to the anterior and posterior sacral region; the fractions of the radiation were 6,000 and 200 centigray. The fraction number was 30. At early postoperative evaluations (one and three months), no local recurrence or distant metastasis was found. The patient went through a radiological examination in the nine-month follow-up, and no local recurrence was detected in the sacrococcygeal region. However, in the abdominal MRI series, suspicious images were detected in the paraaortic and left inferior hepatic region. Positron emission tomography- (PET-) computed tomography (CT) scans detected a paraaortic lymph node, as well as inferior hepatic and superior peritoneal “hot” lesions. The patient returned to the operating room, where all lesions were excised transperitoneally by a general-surgery team. Interestingly, all specimens were histopathologically diagnosed as chordoma. Neither MRI nor PET-CT scans showed any recurrence, either locally or distantly.

## 3. Discussion

Sacral chordomas feature slow-growing, extradural tumors. They occur with atypical clinical findings. Pain is the cardinal symptom, whereas neurological deficits that vary with lesion size, shape, and location are also reported [3]. Pathological materials present with lobules and vacuolated and moderately atypical neoplastic cells that occur across a myxoid stroma separated by fibrous bands.

Chordomas are locally invasive, have a low tendency to metastasize, and have a poor prognosis in long-term follow-up. Metastasis is seen in 5–40% of chordoma cases. Metastasis of chordomas is common in the liver, lung, lymph nodes, peritoneum, and brain. Metastasis can be seen in 20% of chordomas in the clinical follow-up, as in the case with the current patient. Even more importantly, a large number of metastatic chordomas studies were published prior to the use of MRI, and all diagnoses were based on autopsy findings [6]. In addition, since many patients with metastasis as reported in the literature either were asymptomatic metastatic or exhibited only postpartum evidence, these determinations have led researchers to suggest that these may be the metastatic lesions of chordomas. For instance, Sabuncuoglu et al. [6] assert that the incidence of metastasis is 19% a figure that would indeed be newsworthy; in that study, besides distant metastasis in the spine, metastasis was seen also in the lung and liver [6]. In the present case, we found distant metastasis of the peritoneal region. A literature search determines that distant metastasis of the peritoneal region is not frequently detected. Treatment modalities are based on large surgical resections; consequently, successful treatment involves the complete radical resection of the mass. Radiotherapy is advised, to prevent chordoma metastasis. Surgery may require the excision of affected sacral nerve roots, which could in turn result in incontinence, sexual dysfunction, and motor weakness [7]. As a result of large surgical resections, neurological deficits are typically expected postoperatively. No postoperative neurological deficits occurred in our case, given the use of microsurgery. In such cases, survival depends on the successful use of treatment modalities. The median survival rate has been estimated to be approximately six years, with a survival rate of 70% for five years, decreasing to 40% for 10 years [3].

Peritoneal metastasis (also known as peritoneal carcinomatosis, when extensive) is relatively common, particularly in tumors of the abdomen and pelvis; it generally implies a poor prognosis, often with a significant impact on palliation [8, 9]. The most common cancers to invade the peritoneal region are ovarian cancer, gastric cancer, colorectal cancer, appendiceal malignancies, gallbladder carcinoma, pancreatic carcinoma, primary peritoneal malignancy, and hematogenous spread (breast cancer, lung cancer, and malignant melanoma) [9]. Isolated peritoneal metastases are usually asymptomatic. Besides this, peritoneal carcinomatosis may also be asymptomatic, but most patients eventually report symptoms that can range from uncomfortable to debilitating. Symptoms include abdominal distention due to malignant ascites; abnormal bowel motility, resulting in nausea or bloating; intermittent pain; and bowel obstruction. MRI is much more sensitive than CT in diagnosing peritoneal metastasis (i.e., by 85–90%). Peritoneal metastases are not locally treated, although systemic treatment may have some effect [8, 9]. There has been no report in the literature of peritoneal metastasis of chordomas. Aydın et al. [8] report chordoma cells being identified in the peritoneal fluid of patients who had chordomas [8–10]. In the present case, we detected peritoneal metastasis in control MRI and CT scans. There were no symptoms in the patient that were related to peritoneal metastasis. A literature search for peritoneal metastasis treatment in chordomas determines that there are no certain treatment modalities for that phenomenon. The main purpose in such cases is to treat symptoms palliatively.

The standard treatment for chordoma is surgery, but not even surgery can ensure survival; therefore, these patients are usually not completely cured and so most ultimately succumb. No certain surgical technique has been published, but the main purpose of surgical treatment is to facilitate resection as much as possible. Surgical techniques are defined as simple tumor resections and sacrectomy and posterior instrumentations. The posterior approach is mostly suitable for lesions that occur below the third sacral segment [11–13]. We performed a posterior approach to the lesion, as it was below the third sacral segment. For this reason, no combination of surgical techniques and instrumentation was needed.

The size of the surgical resection is a major factor in an asymptomatic survey of these tumors. The surgical dissection must extend to the radiologically normal vertebra. However, these surgical dissections can create some important morbidities, like urine and fecal incontinence. It has been published that lumbosacral stabilizations are required following total sacrectomies [7, 11]. In our case, due to the microdissection technique used, after the nine-month follow-up, there was no neurological deficit and still no need for stabilization.

York et al. report an eight-month disease-free interval following subtotal resection, compared to 2.27 years following radical surgery [13]. The disease-free interval can be longer following a total sacrectomy; however, neurological deficits are more frequently observed. Schoenthaler et al. report 14 patients being treated with helium or neon ions; four of the 14 patients were treated after gross tumor resection, and there was a five-year local control rate of 55% [10]. They also report a trend of better local control in patients treated with neon ions, compared to those treated with helium ions, and for four patients who received RT after gross tumor resection [13]. In our case, after the nine-month follow-up, no local recurrence without radiotherapy and chemotherapy was observed; nonetheless, distant metastasis was detected.

## 4. Conclusion

Chordomas are locally invasive and have a low tendency to metastasize. As in the present case, metastasis may be seen in the clinical follow-up of chordoma cases. In clinical practice, distant metastasis to the peritoneal region is not as common as our case would suggest. The use of radical surgery can determine the efficiency of treatment in sacral chordomas, and it is clear that radical resection of the mass may prevent metastasis.
